# Transcriptional Aneuploidy Responses of *Brassica rapa*-*oleracea* Monosomic Alien Addition Lines (MAALs) Derived From Natural Allopolyploid *B. napus*

**DOI:** 10.3389/fgene.2019.00067

**Published:** 2019-02-13

**Authors:** Zhu Bin, Pan Qi, Huo Dongao, Zeng Pan, Cai Bowei, Ge Xianhong, Li Zaiyun

**Affiliations:** ^1^School of Life Sciences, Guizhou Normal University, Guiyang, China; ^2^National Key Laboratory of Crop Genetic Improvement, National Center of Oil Crop Improvement, College of Plant Science and Technology, Huazhong Agricultural University, Wuhan, China; ^3^Research Center of Buckwheat Industry Technology, Guizhou Normal University, Guiyang, China

**Keywords:** allopolyploid, *Brassica napus*, *Brassica rapa*, monosomic alien addition lines, aneuploidy, transcriptome

## Abstract

Establishing the whole set of aneuploids, for one naturally evolved allopolyploid species, provides a unique opportunity to elucidate the transcriptomic response of the constituent subgenomes to serial aneuploidy. Previously, the whole set of monosomic alien addition lines (MAALs, C1-C9) with each of the nine C subgenome chromosomes, added to the extracted A subgenome, was developed in the context of the allotetraploid *Brassica napus* donor “Oro,” after the restitution of the ancestral *B. rapa* (RBR Oro) was realized. Herein, transcriptomic analysis using high-throughput technology was conducted to detect gene expression alterations in these MAALs and RBR. Compared to diploid RBR, the genes of all of the MAALs showed various degrees of dysregulated expressions that resulted from *cis* effects and more prevailing *trans* effects. In addition, the *trans*-effect on gene expression in MAALs increased with higher levels of homology between the recipient A subgenome and additional C subgenome chromosomes, instead of gene numbers of extra chromosomes. A total of 10 *trans*-effect dysregulated genes, among all pairwise comparisons, were mainly involved in the function of transporter activity. Furthermore, highly expressed genes were more prone to downregulation and vice-versa, suggesting a common trend for transcriptional pattern responses to aneuploidy. These results provided a comprehensive insight of the impact of gene expression of individual chromosomes, in one subgenome, on another intact subgenome for one allopolyploid with a long evolutionary history.

## Introduction

As a deviation from the normal genome, by either gaining or losing entire chromosomes or chromosomal segments, aneuploidy disrupts the genome balance, which generally results in profoundly and severely impaired phenotypes (Antonarakis et al., 2004; Birchler and Veitia, 2007; Huettel et al., 2008; Henry et al., 2010; Siegel and Amon, 2012; Zhu et al., 2015). Some syndromes and diseases, which lead to severe developmental defects and even death in humans, have been associated with variants of entire chromosomes or chromosomal segments (Antonarakis et al., 2004; Morrow, 2010), such as trisomy 21 (Down syndrome) (Letourneau et al., 2014). Moreover, typical chromosomal structural changes, which occur extensively in the majority of solid tumor cells, are believed to induce tumor development (Matzke et al., 2003; Sheltzer and Amon, 2011; Gordon et al., 2012). Since aneuploid studies that involve a single or few chromosomes are limited, genome-wide transcriptional response patterns to aneuploidy remains unclear. Next-generation sequencing, which has been extensively applied to transcriptional analysis, provides a robust means to elucidate gene expression in aneuploid organisms.

Researchers initially believed that phenotypic consequences of aneuploidy are mainly influenced by gene dosage effects of altered chromosomes, which is supported by the correlation between chromosome copy numbers and relative gene expression levels in Down syndrome (Mao et al., 2003), aneuploid yeast (Torres et al., 2008), as well as various types of cancer cells (Gao et al., 2007; Xu et al., 2010; Lu et al., 2011). However, studies on aneuploids in *Drosophila* and plants showed that changes in the gene expression levels were observed along altered copy numbers (*cis*-effects) as well as in unaltered disomic chromosomes (*trans*-effects) (Guo and Birchler, 1994; Birchler and Veitia, 2007; Huettel et al., 2008; Malone et al., 2012; Zhu et al., 2015, 2018; Zhang et al., 2017; Rey et al., 2018). In addition, the phenotypic consequences of aneuploidy are believed to result from either the contribution of altered dosages or the impact of a genome imbalance involving the rest of the genome (Guo and Birchler, 1994; Birchler and Veitia, 2007). Aneuploids exhibit a more severe degree of perturbation in gene expression than changes in ploidy (Birchler and Newton, 1981; Guo et al., 1996), indicating a disruption in the dosage-sensitive gene products (Birchler et al., 2001; Henry et al., 2006).

Plants have better tolerance to chromosomal changes than animals do (Siegel and Amon, 2012). The frequency of aneuploidy increases in polyploids, and the whole set of aneuploids with the loss of each chromosome was established in allohexaploid wheat (Sears, 1954), which may be attributed to a significantly higher capacity to buffer dosage imbalance. Moreover, the aneuploidy with whole-chromosome and structural alterations extensively occurs in nascent allopolyploids (Mestiri et al., 2010; Xiong et al., 2011; Chester et al., 2012; Zhang et al., 2013), implying that aneuploidy is a continuously occurring process until a stable polyploid status is attained. Recent studies have suggested that aneuploidy serves as a rapid mechanism for the adaptation to rare cases of strong selective pressure (Pavelka et al., 2010; Siegel and Amon, 2012).

The whole set of aneuploids in one species is of great value to elucidate the genome structure, relationships and functional interplay from a classical genetic analysis to genome sequencing (Sears, 1954; Mayer et al., 2014; Zhang et al., 2017). However, the establishment of a complete stock of aneuploids is relatively time consuming and has only been achieved for a few species, such as bread wheat (Sears, 1954) and tobacco (Clausen and Cameron, 1944). The allotetraploid *Brassica napus* L. (2n = 4x = 38, AACC), which originated only 7500 years ago from natural hybridization between the ancestors of two extant diploids, *B. rapa* L. (2n = 2x = 20, AA) and *B. oleracea* L. (2n = 2x = 18, CC) (Nagaharu, 1935; Chalhoub et al., 2014), has been used as a model system to investigate the genomic alterations and interactions during allopolyploidization (Albertin et al., 2006; Szadkowski et al., 2010; Zhang et al., 2015). In particular, the ancestral *B. rapa* (RBR Oro) was restituted from natural *B. napus* genotype “Oro” by successively inducing the preferential elimination of the C subgenome chromosomes in intertribal crosses with another crucifer (Tu et al., 2010). Then, by crossing and backcrossing RBR Oro with *B. napus* donor “Oro,” the C subgenome was dissected *in situ* by adding each of its nine chromosomes to RBR Oro and establishing the whole set of *B. rapa*-*oleracea* monosonic alien addition lines (MAALs) (Zhu et al., 2016). These MAALs, with the genetic context of the same natural *B. napus*, provide a unique avenue to decipher the gene expression interaction of two constituent subgenomes under aneuploidy situations.

In view of this, we performed a RNA-Seq to detect the changes in the global gene expression in these nine MAALs, compared to RBR Oro, to reveal transcriptional aneuploidy response patterns associated with the disomic A subgenome chromosomes. The results of this study provide new insights into the impact of individual chromosomes, from one subgenome on another complete subgenome, for one natural allopolyploid.

## Materials and Methods

### Plant Materials

The ancestral *B. rapa* (RBR Oro) was restituted from a natural *B. napus* cultivar “Oro,” by inducing the preferential elimination of the C subgenome chromosomes in intertribal crosses between *B. napus* and another crucifer *Isatis indigotica* (Tu et al., 2010). Then, a complete set of nine *B. rapa-oleracea* MAALs (2n = 21, AA+1C_1-9_) was established to dissect the C subgenome in the A subgenome background through crossing and successive backcrossing between Oro and RBR Oro (Zhu et al., 2016), which was designed, for convenience, as C1-C9. Due to severe self-incompatibility and variable transmission rates of extra chromosomes in MAALs, MAALs plants were pollinated by RBR Oro to generate sufficient progeny seeds. Seedlings were grown in the greenhouse at the Huazhong Agriculture University (Wuhan, China). Fifty progeny plants were established for C1, C2, C3, C5–C8, respectively, and 100 plants for MAALs C4 and C9, due to the much lower transmission rate of these two C subgenome chromosomes via female gametes (Zhu et al., 2016). At least six additional progeny plants for each of the nine MAALs were identified for further analysis.

To screen the additional plants with extra chromosomes of the C subgenome, these progeny plants were first investigated by PCR amplification of the chromosome-specific gene markers that were distributed on both arms of the target chromosomes, and 6 to 21 target plants were identified. Then, a chromosome counting and fluorescence *in situ* hybridization (FISH) analysis with the C subgenome-specific probe (Zhu et al., 2016) was conducted to determine the karyotype integrity of the target plants (Supplementary Figure S1). The ovaries of young flower buds were collected and treated with 2 mM 8-hydroxyquinoline for 3 h at ∼20°C, and then fixed in Carnoy’s solution (3:1 ethanol: glacial acetic acid, v/v) and stored at -20°C for cytological analysis. A cytological observation and FISH analysis was conducted according to Li et al. (1995) and Cui et al. (2012), respectively.

### RNA Extraction and Preparation of cDNA Libraries

Since there were minimal phenotypic differences between RBR and MAAL plants at the three-leaf stage, the third newly expanded leaves, without petioles, from target plants were collected and immediately stored in liquid nitrogen for RNA extraction. Three biological replicates, per MAAL and RBR Oro, were prepared to assess gene expression. Briefly, young leaves from two target plants per MAAL were pooled equally and ground in liquid nitrogen. Total RNA was extracted using a commercial RNA kit (Tiangen, China) according to the manufacturer’s protocol. The quality and quantity of the isolated RNA was assessed using a Qubit (Invitrogen, Life Technologies), and the RNA integrity number (RIN) was then assessed with an Agilent Technologies 2100 Bioanalyzer (Agilent). Samples with RIN < 8 were excluded from further analysis. For the cDNA library construction, 1.5 μg of total RNA per biological replicate was prepared according to the TruSeq RNA Sample Prep v2 protocol (Illumina). Subsequently, 30 libraries were sequenced on an Illumina HiSeq^TM^ 3000 platform (Illumina), to generate 150-bp paired-end reads. The raw sequence data were subjected to data quality control checks based on per base sequence quality scores, per sequence quality scores, per sequence GC content, sequence length distribution, and overrepresented sequences, which are implemented in the FastQC^1^.

### Differentially Expressed Genes (DEGs) Analysis

Trimmomatic version 0.33^2^ (Bolger et al., 2014) was used to remove adapters and low-quality reads, to generate clean reads with the following parameters: LEADING: 3, TRAILING: 3, SLIDINGWINDOW: 4:15, MINLEN: 36, LEADING: 3, TRAILING: 3, SLIDINGWINDOW: 4:15, MINLEN: 36, and TOPHRED: 33. Clean reads were then aligned to the *B. napus* reference genome with 101,040 predicted genes^3^ (Chalhoub et al., 2014) using HISAT version 0.1.6^4^ (Kim et al., 2015). Considering the homologous regions between the A sub-genome and by adding chromosomes of the C subgenome, we performed a strict mismatch tolerance (one-base mismatch) to determine the affiliation of these clean reads according to the following criterion: specifying -L, 0, -0.15, and only using the uniquely mapped reads, in gene expression profiling. The fragments per kilobase per million mapped reads (FPKM) value was calculated using Cufflinks version 2.2.1^5^ with default parameters (-p -G –multi-read-correct) to assess gene expression (Trapnell et al., 2012). To identify differences in gene expression between MAALs and euploid RBR Oro, the package of Deseq2 in the R-project version 3.4.3^6^, with a fold change of at least 2 in the gene expression and false discovery rate of *q* < 0.05 (Trapnell et al., 2013), was employed to identify DEGs. Because one replicate for C3 and C8 showed a low pairwise correlation coefficient (0.44 and 0.67 in C3, 0.47 and 0.65 in C8) with the remaining two replicates, respectively, only two replicates for C3 and C8 were performed to calculate the DEGs. For GO enrichment, because of the lack of gene annotation information in *B. napus*, we used Blast2GO^7^ to retrieve the *B. napus* GO annotation file, by searching the protein database of the GenBank (Conesa et al., 2005). The gene ontology (GO) enrichment analysis was then performed by hypergeometric distribution in R, with an adjusted *p*-value under 0.05 as a cutoff, to determine significantly enriched GO terms (Conesa et al., 2005^8^). Another tool for GO analysis, AgBase GoAnna version 2.00^9^ (McCarthy et al., 2006) was used to annotate the co-regulated *trans*-effected DEGs (adjusted *p* < 0.05).

### Data Statistics and Visualization

To check the statistical significance of the dysregulated expression between each MAAL vs. RBR Oro, a Chi-square test was used with a 0.05 *q*-value as the cutoff. To determine whether the *trans*-acting effects on gene expression were associated with the gene number of extra chromosomes, Pearson correlation coefficient (PCC) with an FDR-adjusted *p*-value < 0.05 as the cutoff was used. To check whether the different expression levels of the genes were similarly impacted by these extra chromosomes of the C subgenome, a Wilcoxon signed-rank test was used with 0.05 *q*-value as the cutoff. Statistical analyses were conducted using the R-project. All of the RNA-Seq data, including raw data and process documentation used in this study is available at the Gene Expression Omnibus (GEO), accession number GSE111510.

Fluorescence *in situ* hybridization images were captured using a computer-assisted fluorescence microscope with a CCD camera (Axio Scope A1, Zeiss, Oberkochen, Germany). Images of the gene expression analysis were generated by plotting functions of the R-project and then composed by Adobe Illustrator version CC.

## Results

### DEGs Between MAALs and RBR

After trimming, to remove adapters and low-quality reads (Q < 20), 7.04–14.76 million clean reads from each replicate were obtainestructural variants at the correspondingd (Supplementary Table S1). Since MAALs and RBR Oro were derived from one natural *B. napus* cultivar, the clean reads for all of the samples were aligned to the *B. napus* reference genome (Chalhoub et al., 2014). In total, 74.27-77.94% clean reads per replicate were obtained, which included 67.58-70.83% unique mapping reads and 6.33-7.83% multiple mapping reads (Supplementary Table S1). Due to the close relationship between the A and C subgenomes, or within the C subgenome chromosomes (Prakash et al., 2009; Chalhoub et al., 2014), a small proportion of the clean reads were aligned to the missing chromosomes in all MAALs and RBR Oro. To reduce the noise of homologous genes, these reads and multiple mapping reads were excluded from further analysis.

e DEseq2 package software was employed to determine DEGs. Compared to RBR Oro, 2,099–3,556 DEGs were identified in nine MAALs (Table 1). Briefly, RBR vs. C1 (3556 DEGs, 3.52%) and RBR vs. C4 (3430 DEGs, 3.39%) showed the highest and comparable DEGs (*χ*^2^ test, *p* = 0.128), followed by RBR vs. C7 (2,959 DEGs, 2.93%), RBR vs. C3 (2,660 DEGs, 2.63%), RBR vs. C2 (2,469 DEGs, 2.44%) and RBR vs. C5 (2,461 DEGs, 2.44%). RBR vs. C6 (2,177 DEGs, 2.15%), RBR vs. C8 (2114 DEGs, 2.09%), and RBR vs. C9 (2,099 DEGs, 2.08%) exhibited the lowest DEGs. Additionally, as expected, due to the existence of extra C subgenome chromosomes, the genes in the MAALs, except C1, remained upregulated (occupying 71.81-86.92% of DEGs) (*χ*^2^ test, *p* < 0.01). However, compared to RBR Oro, C1 presented a higher number of downregulated genes (1,895 DEGs vs. 1,661 DEGs) (*χ*^2^ test, *p* < 0.01), which indicated that the chromosomes of the A subgenome exhibit downregulated gene expression. The details on the DEGs in all pairwise comparisons are listed in Table 1.

**Table 1:** Summary of up- and down-regulated genes in all pairwise comparisons between RBR Oro and MAALs.

Samples	Up regulated genes	Ratio (%)	Down regulated genes	Ratio (%)	Total	Ratio (%)
RBR VS C1	1661	46.71	1895^∗∗^	53.29	3556^A^	3.52
RBR VS C2	1773^∗∗^	71.81	696	28.19	2469^C^	2.44
RBR VS C3	2312^∗∗^	86.92	348	13.08	2660^C^	2.63
RBR VS C4	2524^∗∗^	73.59	906	26.41	3430^A^	3.39
RBR VS C5	2025^∗∗^	82.28	436	17.72	2461^C^	2.44
RBR VS C6	1842^∗∗^	84.61	335	15.39	2177^D^	2.15
RBR VS C7	2352^∗∗^	79.49	607	20.51	2959^B^	2.93
RBR VS C8	1761^∗∗^	83.3	353	16.7	2114^D^	2.09
RBR VS C9	1761^∗∗^	83.9	338	16.1	2099^D^	2.08


*^∗∗^The group of up-/down- DEGs is significantly higher than oppositely regulated genes (chi-square, *p* < 0.01). ^*A,B,C,D*^Different groups were calculated by chi-square (*p* < 0.001).*

### Pairwise Comparisons of *Cis*- and *Trans*-Effects on Gene Expression

In the present study, 14.76-60.78% of the DEGs were directly attributed to the extra chromosomes of the C subgenome (*cis*-effects), and the majority consisted of upregulated genes, primarily due to the genome dosage. However, several downregulated genes on additional chromosomes of the C subgenome were observed in eight MAALs, except for C7, probably as a result of the failure to distinguish noise caused by the high expression of their homeologs in RBR.

Furthermore, after DEGs in the additional chromosome were ruled out, all of the pairwise comparisons of each MAAL vs. RBR Oro indicated widespread *trans*-effected dysregulated genes, but at substantially variable magnitudes (Table 2). Briefly, 686–2,899 DEGs in the A subgenome of the MAALs (occupying 1.54-6.52% of all 44,452 assembled genes, representing 23.04-65.13% of total DEGs) were dysregulated. C1 showed the most widespread *trans*-effects with 2,899 DEGs (6.52% of the total 44,452 assembled genes), followed by C4 (1773 DEGs, 3.99% of the 44,452 assembled genes), C2 (1275 DEGs, 2.87% of the 44,452 assembled genes) and C7 (1147 DEGs, 2.58% of the 44,452 assembled genes), whereas the remaining five MAALs (C6, C5, C3, C8, and C9) exhibited lower but similar *trans*-effects (686–818 DEGs, occupying 1.54-1.84% of the 44,452 total number of assembled genes) (*χ*^2^ test, *p* < 0.001, Table 2).

**Table 2:** *Cis*-and *trans*-effected dysregulated genes in all pairwise comparisons between MAALs and RBR Oro.

Comparisons	*Trans*-effects			*Cis*-effects		
		
	Up-(%)	Down-(%)	Total (%)	Up-(%)	Down-(%)	Total (%)
RBR VS C1	1022 (35.25)	1877 (64.75)^∗∗^	2899 (6.52)^A^	639 (97.26)	18 (2.74)	657 (14.76)
RBR VS C2	581 (45.57)	694 (54.43)^∗∗^	1275 (2.87)^C^	1192 (99.83)	2 (0.17)	1194 (38.77)
RBR VS C3	393 (53.32)	344 (46.68)	737 (1.66)^E,F^	1919 (99.79)	4 (0.21)	1923 (60.78)
RBR VS C4	871 (49.13)	902 (50.87)	1773 (3.99)^B^	1653 (99.76)	4 (0.24)	1657 (35.83)
RBR VS C5	336 (43.58)	435 (56.42)^∗∗^	771 (1.73)^E,F^	1689 (99.94)	1 (0.06)	1690 (50.49)
RBR VS C6	485 (59.29)^∗∗^	333 (40.71)	818 (1.84)^E^	1357 (99.85)	2 (0.15)	1359 (50.67)
RBR VS C7	540 (47.08)	607 (52.92)^∗^	1147 (2.58)^D^	1812 (100.00)	0	1812 (50.04)
RBR VS C8	334 (48.69)	352 (51.31)	686 (1.54)^F^	1427 (99.93)	1 (0.07)	1428 (56.60)
RBR VS C9	369 (52.34)	336 (47.66)	705 (1.59)^F^	1392 (99.86)	2 (0.14)	1394 (52.07)


*^*A,B,C,D,E,F*^Different groups were calculated by chi-square (*p* < 0.001). ^∗∗^The group of up-/down-DEGs is significantly higher than oppositely regulated genes (*χ*^*2*^, ^∗^*p* < 0.05, ^∗∗^*p* < 0.01).*

### *Trans*-Effect Dysregulation Genes Not Associated With the Gene Number of Extra Chromosomes, and Different Transcriptional Responses of Disomic Chromosomes to Aneuploidy

Analysis of the Pearson correlation coefficient (PCC) did not detect any correlations (*R*^2^ = 0.11, *p* = 0.39) between the number of genes along the additional C chromosomes and corresponding dysregulated genes on the A subgenome. Furthermore, no strong relationship between the expressed genes (FPKM > 0.1) of the extra chromosomes of the C subgenome and changes in gene expression profiles in the A subgenome, were observed (*R*^2^ = 0.27, *p* = 0.15). Inversely, the gene expression along each chromosome of the A subgenome was distinctly impacted by different additional chromosomes in MAALs, as the extent of the *trans*-effects on each of its 10 chromosomes, were uneven in all comparisons except for RBR vs. C3, and the genes on some chromosomes were more prone to transcriptional perturbation, indicating that the identity of additional chromosomes could affect gene expression throughout the A subgenome in a specific manner (Supplementary Table S2). However, MAAL C3 exhibited a similar change in the expression of genes in the A subgenome chromosomes (1.10-2.03% of the genes were dysregulated). To further test the relationship between *trans*-effects on the A subgenome and extra chromosomes of the C subgenome, we then calculated the PCC values of all of the genes in the A subgenome, between any two of the nine MAALs and RBR Oro, and screened for pairwise transcriptome differences (Supplementary Table S3). Compared with other MAALs, C1 always showed significantly lower pairwise correlation coefficients, followed by C2 (Supplementary Table S3). As the homoeologous pairs (A1/C1 and A2/C2) showed the most extensive homoeology, which could frequently be replaced and compensated (Xiong et al., 2011; Chalhoub et al., 2014), we concluded that a higher similarity between the additional chromosome and the recipient genome (chromosome) induced more severe changes in gene expression of the recipient genome.

**FIGURE 1:**
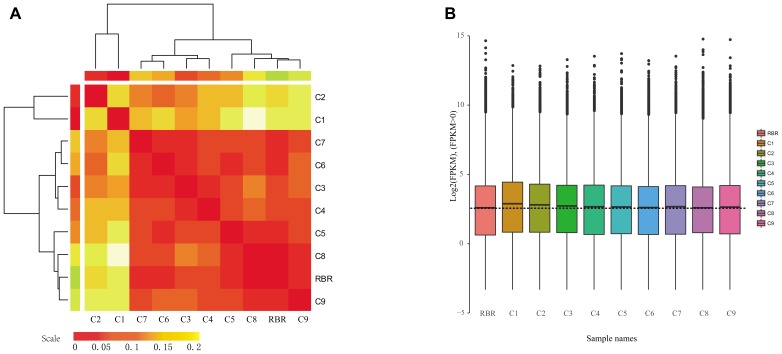
Impact of different additional chromosomes on gene expression patterns in the A subgenome. **(A)** Hierarchical analysis of the global average of all of the gene expression differences in the A subgenome shows that different additional chromosomes result in distinct impacts on gene expression perturbation in the A subgenome, and a higher level of similarity of gene content between the remainder genome and the additional chromosome likely gives rise to greater divergence in gene expression. Two major groups of clusters are identified. **(B)** Box plot of gene expressions along the A subgenome (FPKM > 0.1) also demonstrates that the similarity between the remaining genome and the additional chromosome affects the gene expression levels of the A subgenome in MAALs. The dotted line represents the median of gene expression of the A subgenome in RBR. The *y* axis represents the log_2_(FPKM).

The differential impacts of different additional chromosomes on gene expression in the A subgenome were also reflected by a hierarchical analysis of the similarities in terms of overall expression patterns (Figure 1A). The differences of a global average of all gene expressions showed that MAALs C1 and C2 were most divergent in the overall expression patterns from the rest, which was in line with the result of the pairwise PCC values. Furthermore, the two MAALs were clustered together into an independent group (Figure 1A). Box plots of all expressed gene profiles (FPKM > 0.1) on the recipient A subgenome, in all of the pairwise comparisons, showed that medians of the gene expressions in MAALs C1 and C2 were far from that in RBR Oro and other MAALs, confirming the observed divergence in gene expression (Figure 1B). In addition, the remaining seven MAALs and RBR Oro were divided into another group (Figure 1A). It was determined that MAAL C9 was closely related to RBR Oro and a subgroup was formed. It did however, exhibited a divergent phenotype from RBR Oro (Zhu et al., 2016).

Another interesting result was that among these *trans*-effected DEGs in the pairwise comparisons of each MAAL vs. RBR Oro (Table 2), only the DEGs in RBR vs. C6 showed significantly enhanced expression (*χ*^2^ test, *p* < 0.01). Considering that C6 was the only MAAL that flowered earlier than RBR Oro (Zhu et al., 2016), we questioned whether flowering genes constituted a large fraction of genes that were found to be highly expressed. However, only three genes related to the flowering time in *B. napus*, from the public Brassica Database^10^ were dysregulated, including one downregulated (BnaA03g39820D) and two upregulated DEGs (BnaA01g15350D and BnaA07g13990D).

### Putative Functions of Differently Regulated Gene Groups

**FIGURE 2:**
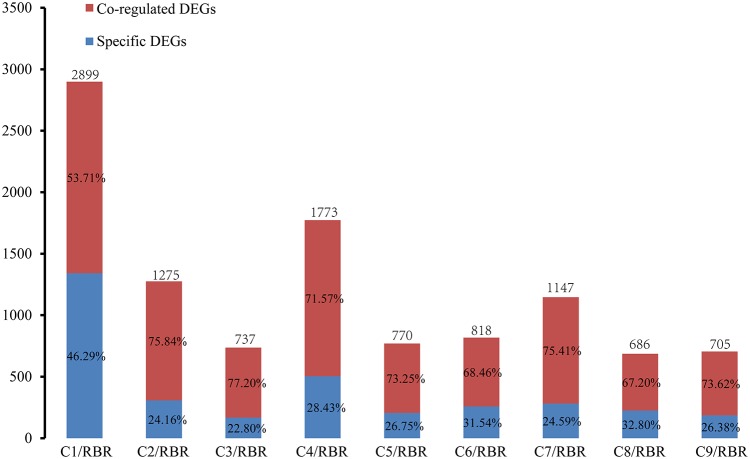
Frequency of specific DEGs and co-regulated DEGs in the A subgenome in all pairwise comparisons. The red box represents the proportion of co-regulated *trans*-effect DEGs that are detected in at least two pairwise comparisons. The blue box represents the proportion of specific *trans*-effect DEGs uniquely observed in a comparison. The *y* axis represents the number of DEGs.

To determine whether some core genes or biological pathways of the recipient A subgenome in MAALs responded to additional chromosomes, Venn diagrams were constructed to determine the specific DEGs and co-regulated DEGs. Due to the ambiguous results in one diagram and because of too many pairwise comparisons, the Venn diagrams were divided into four sections, including three groups based on the results of divergent gene expressions and one group comprising of the co-dysregulated genes from the former three groups (Supplementary Figure S2). Each pairwise comparison uniquely detected 22.80-46.29% of *trans*-effected DEGs (Figure 2). Among these pairwise comparisons, RBR vs. C1 had a significantly higher number of specific DEGs (1,342 DEGs, occupying 46.29% of *trans*-effected DEGs) (*χ*^2^ test, *p* = 2.60e-10), followed by RBR vs. C8 (225 DEGs, 32.80%), RBR vs. C6 (206 DEGs 31.54%), and RBR vs. C4 (504 DEGs, 28.43%), whereas the remaining five MAALs were within the lower ranges of 22.80-26.75%. However, only 10 *trans*-effected dysregulated genes, including one downregulated gene and nine upregulated genes, were always observed in all pairwise comparisons. These results indicate that, despite the uniformity of the global transcriptional aneuploidy response patterns, there was no significant overlap in individual dysregulated genes between different pairwise comparisons of each MAAL vs. RBR Oro. To elucidate whether these 10 co-dysregulated genes were involved in one or specific biological pathway(s), in response to the consequences of aneuploidy on gene expression, we annotated these co-dysregulated genes into the public Gene Ontology (GO) database. However, these genes failed to be clustered into any GO terms or KEGG pathways, probably due to the low number of genes used in the analysis. Therefore, we had to deduce the putative functions of the co-dysregulated genes (Table 3). According to their ortholog gene functions in *A. thaliana*, the upregulated DEGs were mainly involved in “copper ion binding,” “transporter activity,” as well as “protein transporter activity,” implying that MAALs responded to stress by increasing their levels of gene expression. In addition, the downregulated DEG was involved in “rRNA binding” and “structural constituent of ribosome,” which agreed with the reported decrease in protein synthesis in aneuploids (Sheltzer et al., 2012; Stingele et al., 2012).

**Table 3:** The putative gene functions of co-dysregulated genes in all pairwise comparisons of MAALs vs. RBR Oro.

Gene_ID	Up/down	Putative orthologs in *A. thaliana*	Gene functions
BnaA01g13280D	Up	AT4G23600	Cystathionine beta-lyase activity, lyase activity, pyridoxal phosphate binding, transaminase activity
BnaA03g07040D	Up	AT5G18370	Disease resistance protein
BnaA10g17050D	Up	AT5G17700	Antiporter activity, drug transmembrane transporter activity, transporter activity
BnaA07g05110D	Up	AT3G62750	Beta-glucosidase activity, hydrolase activity, hydrolyzing O-glycosyl compounds, scopolin beta-glucosidase activity
BnaA10g14500D	Up	AT5G21100	Copper ion binding
BnaA03g06790D	Up	AT5G17920	Copper ion binding, protein binding
BnaA03g07050D	Up	AT4G24840	Protein transporter activity
BnaA01g28190D	Up	AT3G16150	Asparaginase activity, beta-aspartyl-peptidase activity
BnaA10g21850D	Up	AT5G10420	Antiporter activity, drug transmembrane transporter activity
BnaA07g32060D	Down	AT1G75350	rRNA binding, structural constituent of ribosome


**FIGURE 3:**
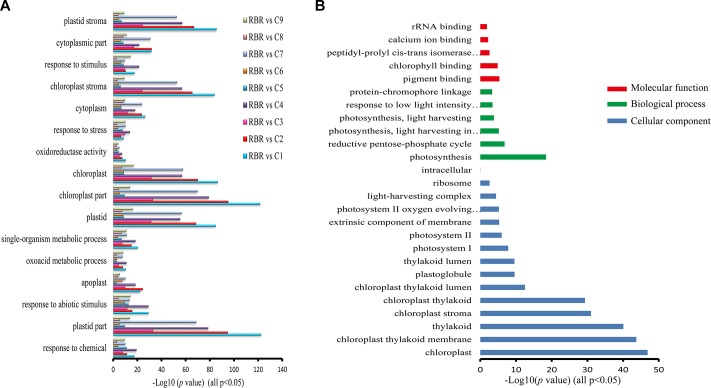
GO enrichments for co-regulated DEGs in all pairwise comparisons and DEGs identified in more than four comparisons. **(A)** A total of 17 GO terms that are particularly involved in biotic and abiotic stresses are enriched with DEGs in all pairwise comparisons (FDR, *p* < 0.05). The *x* axis represents the value of –log_10_(*p-*value). **(B)** GO analysis of DEGs that are identified in more than four pairwise comparisons exhibiting these DEGs are predominantly associated with photosynthetic process and protein synthesis. The *x* axis represents the value of –log_10_(*p-*value).

We then performed gene annotation to investigate the functions of specific and co-regulated DEGs that were detected in at least two pairwise comparisons in all pairwise comparisons, to reveal whether these different gene groups were involved in distinct or similar biological pathways. We found that 17 GO terms were enriched with these co-regulated DEG groups in all comparisons (*p* < 0.05, Figure 3A) and were mainly involved in responses to stress (all *p* < 0.0007), abiotic stimulus (all *p* < 5.91e^-5^) and oxidoreductase activity (all *p* < 0.003), particularly in the chloroplast (all *p* < 4.12e^-8^). As expected, the GO terms enriched by the specific DEGs for each pairwise comparison did not significantly overlap with each other. No GO terms that were enriched in all of the comparisons were observed, and very few terms were detected in most of the comparisons. Since DEGs that were often perturbed in many pairwise comparisons probably represented common features of aneuploidy, 650 *trans*-affected DEGs were identified in more than four pairwise comparisons. The AgBase GoAnna website tool was then employed to analyze the gene ontology annotation of these DEGs (McCarthy et al., 2006). The majority of GO terms (Figure 3B) enriched with these DEGs were associated with a photosynthetic process, such as “photosynthesis” (*p* < 3.81e^-19^), “photosynthesis, light harvesting” (*p* < 0.00015), “chloroplast” (*p* < 1.33e^-47^), and “chlorophyll binding” (*p* < 1.38e^-5^). The terms of “rRNA binding” (*p* < 0.05) and “ribosome” (*p* < 0.0027) were also detected, suggesting that additional C chromosomes generally had a consequence on protein synthesis. Altogether, these results indicated that dysregulated genes, in all comparisons belonging to distinct groups, were involved in diverse cellular pathways.

### Highly Expressed Genes Are Prone to Downregulation and Lowly Expressed Genes to Upregulation

**FIGURE 4:**
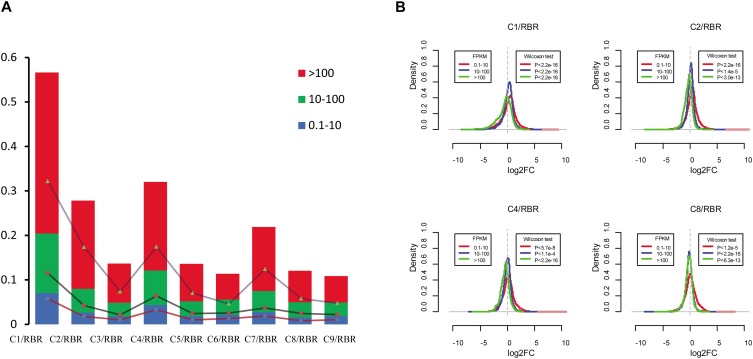
Impact of additional C subgenome chromosomes on changes in gene expression changes in the A subgenome. **(A)** Proportions of *trans*-effect DEGs in different gene expression levels. Based on the gene expression level, genes in the A subgenome are divided into three categories: low-expression level genes (0.1 < FPKM < 10), medium-expression level genes (10 < FPKM < 100), and high-expression level genes (FPKM > 100). For all pairwise comparisons, dysregulated genes consistently show the highest frequency in the high-expression level group, second in the medium-expression level group, and last in the low-expression level group. The *y* axis represents the frequency of the gene groups. **(B)** Frequency distribution of log_2_(fold change) in four MAALs indicate differences in transcriptional response patterns to aneuploidy in various gene expression groups. High-expression level genes are more prone to be downregulated while the opposite is true for low-expression level genes (Wilcoxon test, *q* < 0.05). The *x* axis represents the log_2_(fold-change) in gene expression levels between the MAALs and euploid RBR in three gene groups and the *y* axis represents frequency distribution of log_2_(fold-change).

To analyze the transcriptional aneuploidy response patterns in MAALs, we detected whether the different expression levels of the genes were similarly impacted by these extra chromosomes of the C subgenome. Based on the gene expression level (FPKM > 0.1) in RBR Oro, we classified the genes of the A subgenome into three groups according to low (0.1 < FPKM < 10), medium (10 < FPKM < 100), and high (FPKM > 100) expression levels. About two thirds of the expressed genes (16,714 genes, representing 62.53% of the genes with RPKM > 0.1) belonged to the first group, nearly one third of these (8,699 genes, 32.55%) were in the medium expression level group, and relatively few genes (1,315 genes, 4.92%) were in the high expression level group. Compared to RBR Oro, the lowly expressed genes group gave rise to the lowest proportion of *trans*-effected DEGs (consisting of 1.58-7.04% of the total number of low expression genes with the average of 2.88%), indicating that the lowly expressed genes group was less susceptible to the impact of extra chromosomes of the C subgenome. Both the highly and moderately expressed genes exhibited escalating contributions to the *trans*-effected DEGs (comprising 3.03-13.39% of the medium-level expression genes with an average of 5.29% and occupying 5.78-36.2% of high expression genes with an average of 14.04%), suggesting that these genes were more prone to transcriptional perturbation in aneuploidy (Supplementary Table S4 and Figure 4A).

Intriguingly, it was observed that upregulated genes predominantly accumulated in lowly expressed genes for all pairwise comparisons (*χ*^2^ test, *p* < 0.05), whereas the downregulated DEGs were more prevalent in the medium expression and the high expression level (*χ*^2^ test, *p* < 0.05) groups (Supplementary Table S4). These findings probably indicated some transcriptional patterns in response to extra chromosomes of the C subgenome. To substantiate this hypothesis, we compared the gene expression fold-change distributions of all of the pairwise comparisons for each group, with the expected distribution exhibited by RBR Oro (Supplementary Figure S3 and Figure 4B). As expected, the highly and moderately expressed genes (except in C7, Wilcoxon signed-rank test, *p* = 0.88) were generally biased toward the reduced expression in MAALs (all *p* < 0.05). However, the low expression level genes showed increased expression in all MAALs (Figure 4A). This result agreed with the observation that highly expressed genes contributed substantially to the downregulated and lowly expressed genes, mainly associated with upregulation in a pair of monozygotic twins, discordant for trisomy 21 (Letourneau et al., 2014), suggesting that there might be a common feature in transcriptional pattern responses to aneuploidy.

### No Obvious Dysregulation Domains in MAALs

**FIGURE 5:**
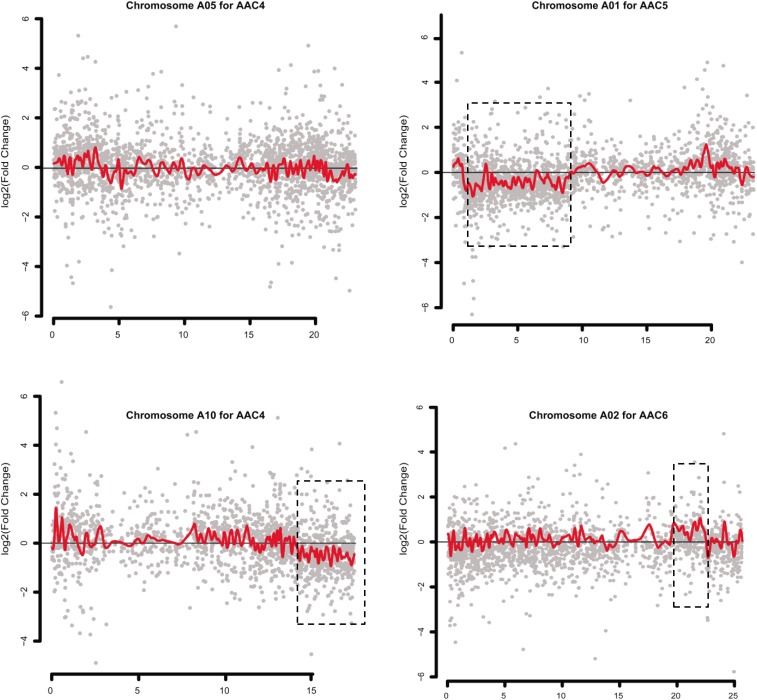
Dysregulated domains of gene expression in different chromosomes in some pairwise comparisons. Log_2_(fold-change) value in the gene expression between one of the MAALs and RBR in the chromosomes is depicted in gray. Red lines denote the smoothed distribution for the differentially expressed genes in these selected chromosomes, as representatives, using the Lowess function of R. Dysregulated domains that are exhibited in dotted boxes are clearly observed in only three chromosomes in three pairwise comparisons (chromosome A1 in C5/RBR, chromosome A10 in C4/RBR and chromosome A02 in C6/RBR). The *y* axis represents the log_2_(fold change) value of FPKM between the MAALs and euploid RBR. The *x* axis represents the sorted positions of genes on these chromosomes.

Gene expression dysregulation domains (GEDDs), wherein dysregulated genes were clustered as domains on chromosomes, were reported in human trisomy 21 (Letourneau et al., 2014) and a nullisomics in *B. napus* (Zhu et al., 2015). In the present study, we smoothed the distribution of gene expression fold changes (log2 ratios) along each normal chromosome for each MAAL, using the Lowess function of R to identify potential GEDDs. Unexpectedly, only three dysregulated regions on different chromosomes were observed in different comparisons (Figure 5), indicating that these GEDDs might not be a common feature for aneuploidy. Furthermore, we couldn’t exclude the possibility that these three dysregulation domains resulted from macroscopic structural variants at the corresponding chromosomes.

## Discussion

The advent of high-throughput sequencing technologies has facilitated the analysis of genome-wide gene expressions involving aneuploids. Recently, numerous studies demonstrated that dysregulated genes in aneuploidy are not only restricted to altered chromosome but also disomic chromosomes (Huettel et al., 2008; Makarevitch and Harris, 2010; Letourneau et al., 2014; Zhu et al., 2015, 2018; Zhang et al., 2017), suggesting that this dysregulation in gene expression may be a collective transcriptional response to aneuploidy (Sheltzer et al., 2012). Phenotypic consequences in aneuploidy might result from *cis*-effects or *trans*-effects or global alterations of the entire regulatory system (Prestel et al., 2010). This theory of genome imbalance involves disruptions in the dosage-sensitive gene products (Birchler et al., 2001; Henry et al., 2006). It is therefore essential to detect whether changes in transcriptional expression are in direct proportion to alterations in the DNA copy number, or whether dosage compensation minimizes the effects of aneuploidy. Our results clearly showed that when compared to parental RBR, all of the MAALs exhibited prevalent *trans*-effect gene expression. However, no strong correlations were observed between *trans*-effects and levels of the chromosome genes number or the expressed gene number. Intriguingly, the MAALs harboring chromosome C1 or C2, showed a significantly higher number of *trans*-effect dysregulated genes (Table 2) and also provided the closest transcriptional responses based on gene expression divergence (Figure 1A). From the extensive homoeology between chromosome pairs (A1/C1 and A2/C2) (Chalhoub et al., 2014), we proposed that the levels of transcriptional responses to aneuploidy were associated with the similarity of the gene content between the remainder of the genome and the altered chromosome(s), rather than with the extent (gene number) of gene alterations. Another intriguing observation was that significantly different levels of gene expressions were observed in the recipient A subgenome, in response to different extra chromosomes of the C subgenome, wherein a higher number of dysregulated genes were observed in more similar chromosomes, such as A1 and A2, than those of other chromosomes.

A landmark study on gene expression from aneuploidy cells in diverse organisms demonstrated that aneuploids of various chromosomes and in different organisms, impacted similar cellular pathways and resulted in stereotypical anti-proliferative responses (Sheltzer et al., 2012). Although different pairwise comparisons between MAALs revealed no significant overlap of individual dysregulated genes in the A subgenome and only 10 co-regulated genes were detected, their functions were clearly involved in either negative proliferation or enhanced stress. GO analysis of co-regulated DEGs, which were identified in more than one comparison, also suggested that similar cellular pathways were related to changes in transcription profiles due to *trans*-effects.

In our study it was observed that highly expressed genes, on disomic chromosomes of the A subgenome, were more prone to transcriptional perturbation in all pairwise comparisons. Furthermore, the set of highly expressed genes seemed to be downregulated in response to aneuploidy. Inversely the set of naturally lowly expressed genes was likely upregulated. A similar phenomenon has been observed in one *B. napus* nullisomics, that lost the chromosome C2 (Zhu et al., 2015) as well as in monozygotic twins discordant for trisomy 21 (Letourneau et al., 2014). Interestingly, in these monozygotic twins, either upregulated or downregulated genes, between the twins, were clustered in the domains along all of the chromosomes (GEDDs), in which the highly expressed genes were mainly distributed in downregulated domains while lowly expressed genes were distributed in upregulated domains (Letourneau et al., 2014).

It was speculated that these dysregulation domains were associated with some phenotypic features of trisomy 21 (Letourneau et al., 2014; Pope and Gilbert, 2014), or that they could be the result of extra chromosomal material (Letourneau et al., 2014). Similar dysregulation domains observed in nullisomic *B. napus* likely supported the latter hypothesis (Zhu et al., 2015), although a transcriptome analysis of trisomy 5 in *Arabidopsis thaliana* (Huettel et al., 2008) and a series aneuploidy in hexaploid wheat (Zhang et al., 2017) did not detect these domains. These results, however, were derived from aneuploid organisms involving only one or a few chromosomes. Among our nine MAALs, only three dysregulated regions on different chromosomes were observed in three comparisons, indicating that the dysregulation domains were likely specific to particular aneuploid organisms.

Altogether, transcriptomic analysis of MAALs provided a comprehensive insight into transcriptional aneuploidy response patterns and allowed a deeper understanding of the functional interplay between the intact A subgenome and the extra C subgenome chromosomes in the natural allopolyploid *B. napus*.

## Author Contributions

ZB designed this study and wrote the manuscript. PQ analyzed the RNA-Seq data. ZP, CB, and HD phenotyped and identified parental RBR “Oro” and MAALs. GX provided the analysis of FISH for all materials. LZ edited the manuscript. All authors have commented and approved the final manuscript.

## Conflict of Interest Statement

The authors declare that the research was conducted in the absence of any commercial or financial relationships that could be construed as a potential conflict of interest.

## Footnotes

1 http://www.bioinformatics.babraham.ac.uk/projects/fastqc/

2 http://www.usadellab.org/cms/index.php?page=trimmomatic

3 http://www.genoscope.cns.fr/brassicanapus/

4 http://www.ccb.jhu.edu/software/hisat/

5 http://cufflinks.cbcb.umd.edu/

6 https://www.r-project.org/

7 https://www.blast2go.com/

8 https://academic.oup.com/bioinformatics/article/21/18/3674/202517

9 http://agbase.arizona.edu/cgi-bin/tools/GOanna.cgi

10 http://brassicadb.org/brad/index.php

## Supplementary Material

FIGURE S1 Identification of target plants in progeny populations of MAALs. **(A)** FISH using a C genome-specific probe demonstrates the target plant with the chromosome complement of 20A+1C. Blue indicates the DAPI counterstaining of chromosomes, and the red signal indicates the labeled BAC BoB014O06 probe specifically for the C genome chromosome (Bar, 10 mm). **(B)** PCR amplification of four chromosome-specific gene primers for the two arms of chromosome C1 to screen the backcrossing population of MAAL C1. The results show that these target plants harbor an integrated chromosome C1.

FIGURE S2 Venn diagram of *trans*-effect DEGs in all pairwise comparisons. Due to the ambiguity of all nine pairwise comparisons in one picture, we divide the nine comparisons into three groups based on the results of a hierarchical analysis of gene expression divergence.

FIGURE S3 Frequency distribution of log_2_(fold change) values in all MAALs. High-expression level genes are more prone to be downregulated while the opposite is true for low-expression level genes in all pairwise comparisons (Wilcoxon test, *q* < 0.05). The *x* axis represents the log_2_(fold change) value in gene expression between the MAALs and euploid RBR in the three gene groups, and the *y* axis represents the frequency distribution of log_2_(fold-change).

TABLE S1 Summary of clean reads of MAALs and RBR Oro aligned to the reference genome (*B. napus*).

TABLE S2 Uneven distribution of DEGs of *trans*-effects across all chromosomes in each of the comparisons between RBR and the serial MAALs.

TABLE S3 Pairwise Pearson correlation coefficient (PCC) values of transcriptomic differences among all genes in the A subgenome between any two of the nine MAALs and RBR (The FPKM values of all 44,452 genes in the A genome per sample is used as the dataset, then the PPC values of these datasets are calculated using the correlation function of the R-project).

TABLE S4 The distribution of dysregulated genes in different gene expression levels.

## Abbreviations

DEGs: differentially expressed genes
FISH: fluorescence *in situ* hybridization
GEDDs: gene expression dysregulation domains
MAAL: monosomic alien addition line
RBR: restituted *B. rapa*
