# Supplementary figures and images for: Transcriptional Aneuploidy Responses of Brassica rapa-oleracea Monosomic Alien Addition Lines (MAALs) Derived From Natural Allopolyploid B. napus
Source: Front Genet. 2019 Feb 13;10:67. doi: 10.3389/fgene.2019.00067 (PMC6381038; doi:10.3389/fgene.2019.00067)

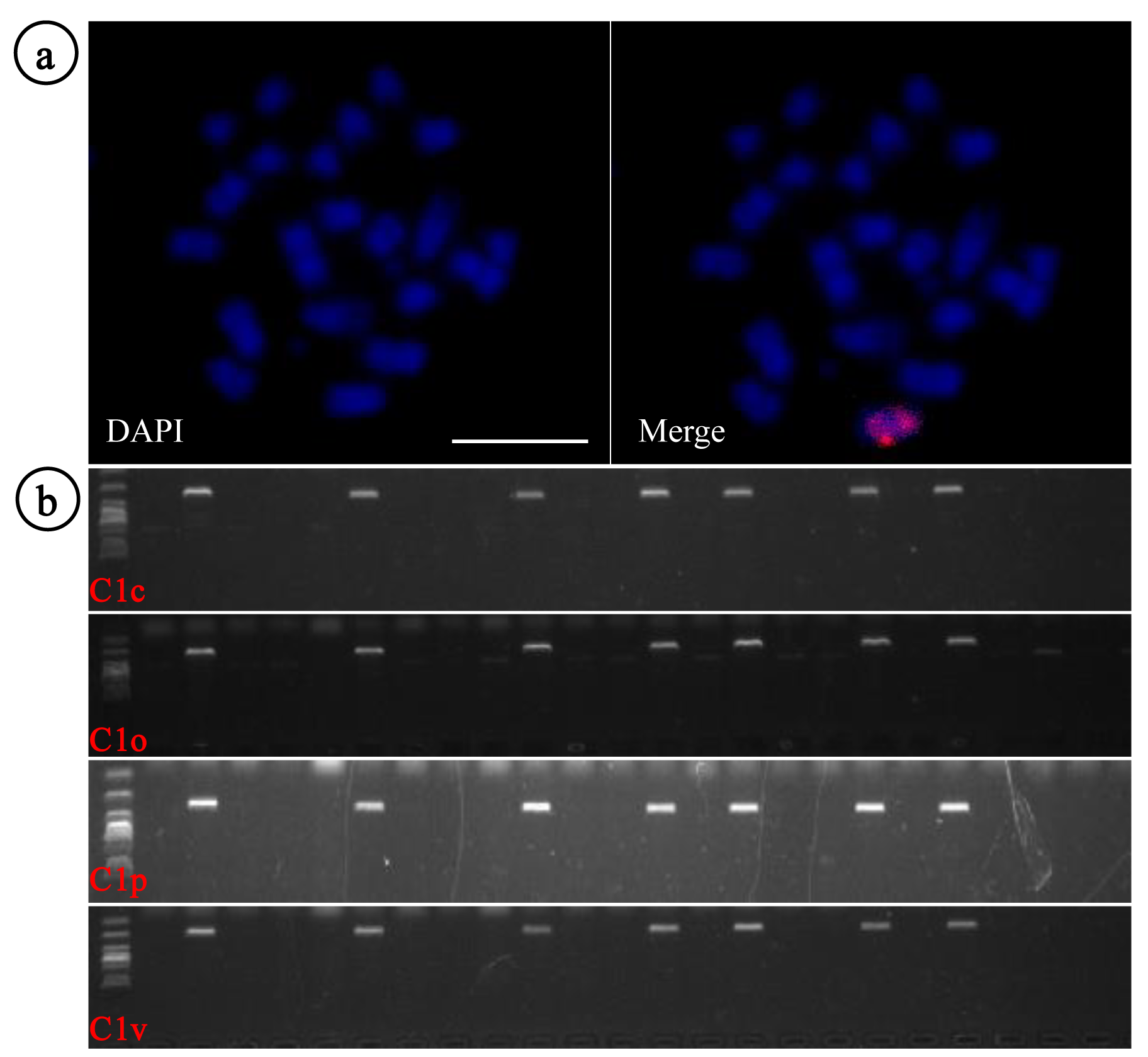

Supplement: FIGURE S1 — Identification of target plants in progeny populations of MAALs. (A) FISH using a C genome-specific probe demonstrates the target plant with the chromosome complement of 20A+1C. Blue indicates the DAPI counterstaining of chromosomes, and the red signal indicates the labeled BAC BoB014O06 probe specifically for the C genome chromosome (Bar, 10 mm). (B) PCR amplification of four chromosome-specific gene primers for the two arms of chromosome C1 to screen the backcrossing population of MAAL C1. The results show that these target plants harbor an integrated chromosome C1. [file Image_1.TIF]

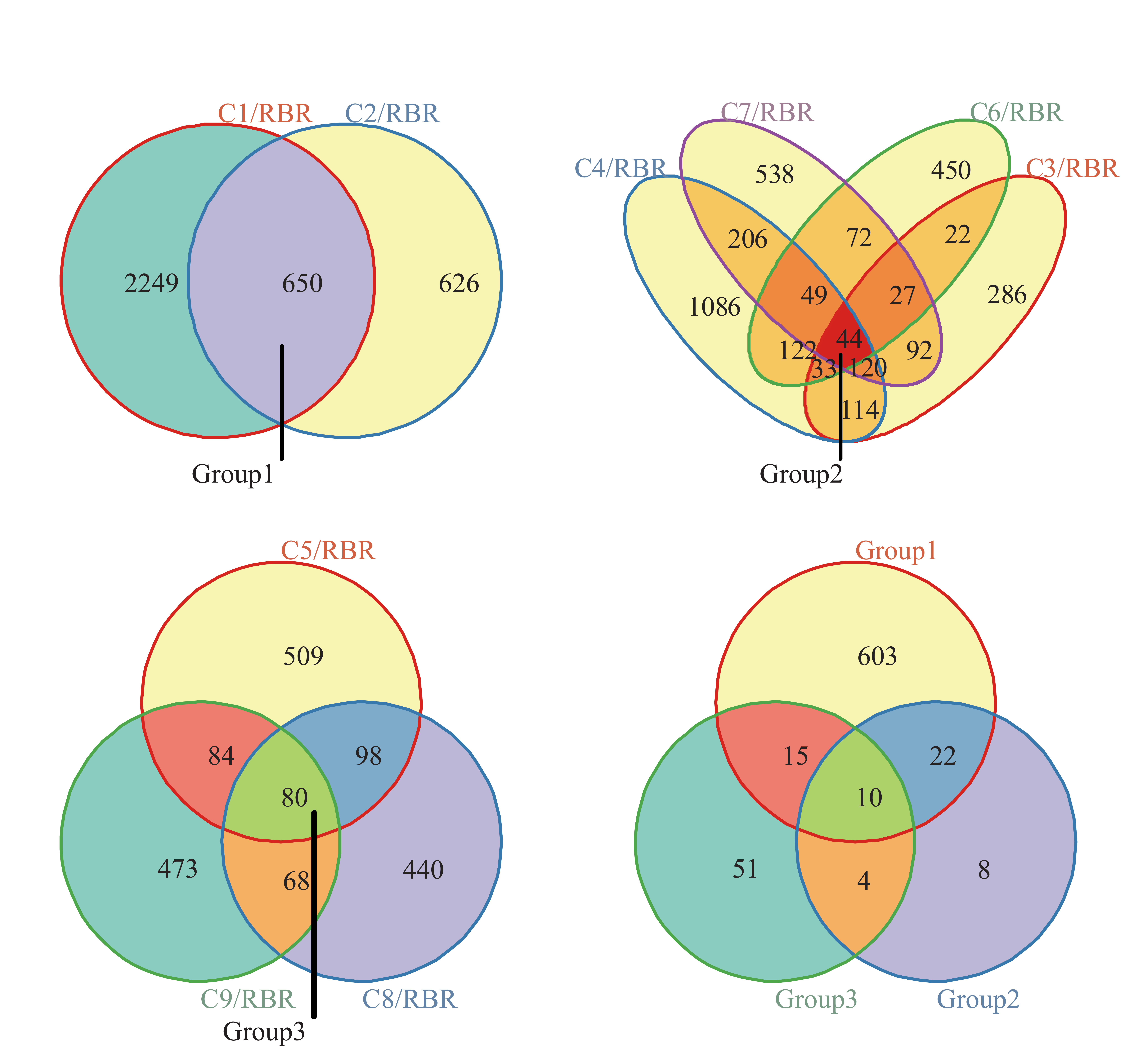

Supplement: FIGURE S2 — Venn diagram of trans-effect DEGs in all pairwise comparisons. Due to the ambiguity of all nine pairwise comparisons in one picture, we divide the nine comparisons into three groups based on the results of a hierarchical analysis of gene expression divergence. [file Image_2.TIF]

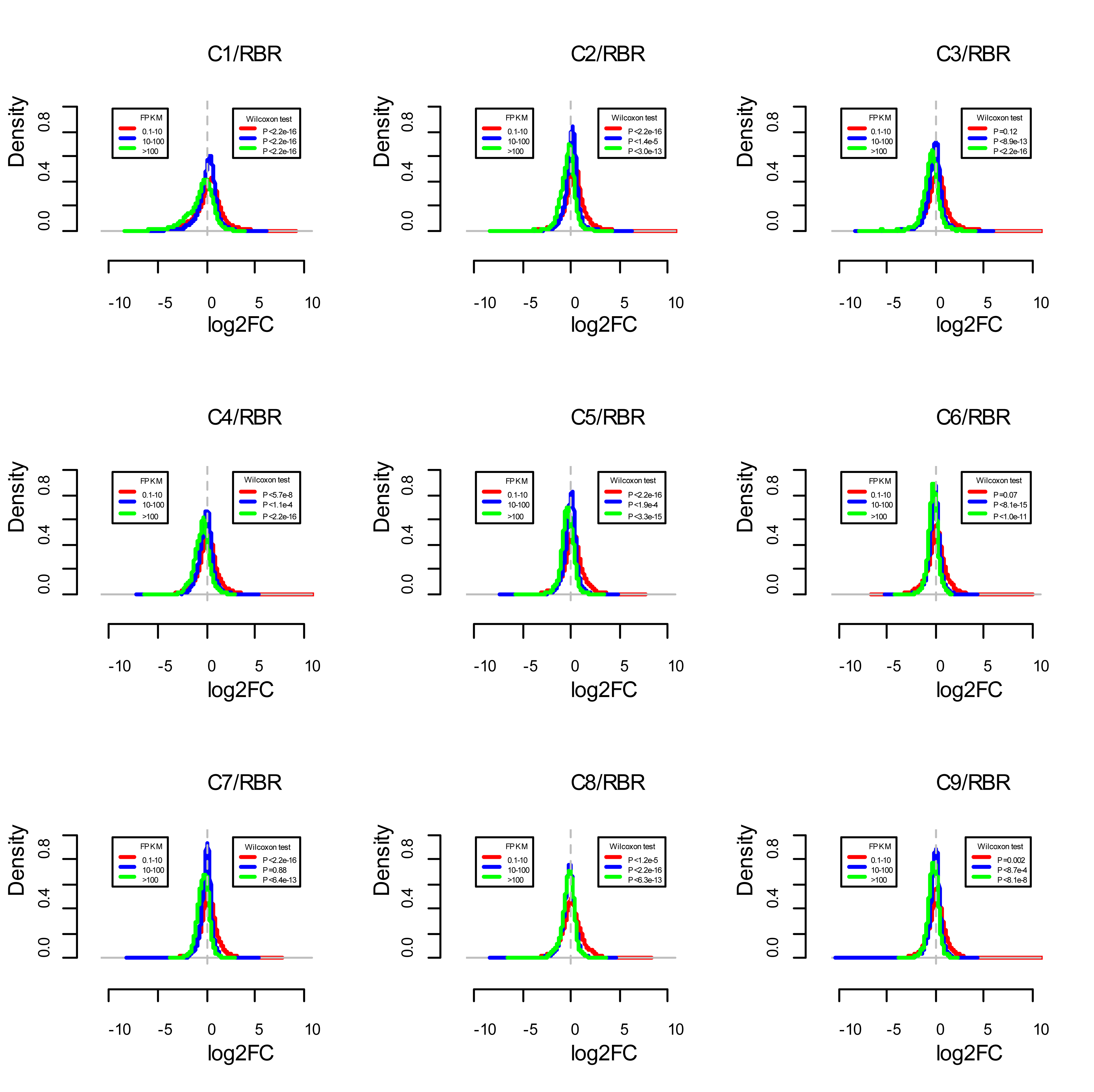

Supplement: FIGURE S3 — Frequency distribution of log2(fold change) values in all MAALs. High-expression level genes are more prone to be downregulated while the opposite is true for low-expression level genes in all pairwise comparisons (Wilcoxon test, q < 0.05). The x axis represents the log2(fold change) value in gene expression between the MAALs and euploid RBR in the three gene groups, and the y axis represents the frequency distribution of log2(fold-change). [file Image_3.TIF]
